# Reduction of Sniff Nasal Inspiratory Pressure (SNIP) as an Early Indicator of the Need of Enteral Nutrition in Patients with Amyotrophic Lateral Sclerosis

**DOI:** 10.3390/brainsci11081091

**Published:** 2021-08-20

**Authors:** Stefano Zoccolella, Rosa Capozzo, Vitaliano N. Quaranta, Giorgio Castellana, Lorenzo Marra, Vito Liotino, Vincenza Giorgio, Isabella L. Simone, Onofrio Resta, Marco Piccininni, Rosanna Tortelli, Giancarlo Logroscino

**Affiliations:** 1 ASL Bari, San Paolo Hospital, Neurology Unit, 70123 Bari, Italy; stefzoc@hotmail.it; 2 Center for Neurodegenerative Diseases and the Aging Brain, University of Bari “Aldo Moro”–A.O. Pia Fond “Card. G. Panico” Hospital, 73039 Tricase, Italy; rosacapozzo@gmail.com (R.C.); giancarlo.logroscino@uniba.it (G.L.); 3 Respiratory and Sleep Disorders Unit, Cardio-Thoracic Department, Policlinic University Hospital, UNIBA, 70124 Bari, Italy; vitalianoquaranta@gmail.com (V.N.Q.); lorenzomarr@gmail.com (L.M.); vitoliotin@gmail.com (V.L.); onofriorest@gmail.com (O.R.); 4 Pulmonary Division, Istituti Clinici Scientifici Maugeri SpA SB Pavia, IRCCS, 70124 Bari, Italy; giorgiocastellan@gmail.com; 5 “L. Fallacara” Hospital, 70019 Triggiano, Italy; vincenzagiorg@gmail.com; 6 Department of Basic Medical Science and Sense Organs, University of Bari “Aldo Moro”, 70124 Bari, Italy; isabella.simone@uniba.it; 7 Institute of Public Health, Charité—Universitätsmedizin Berlin, 10117 Berlin, Germany; marco_piccininni@libero.it

**Keywords:** amyotrophic lateral sclerosis, percutaneous endoscopic gastrostomy, sniff nasal inspiratory pressure, prognosis

## Abstract

Percutaneous endoscopic gastrostomy (PEG) is the standard procedure for feeding severely dysphagic patients with amyotrophic lateral sclerosis (ALS). It is associated with prolonged survival and improvement in quality of life. Nasal inspiratory pressure during a sniff (SNIP) is a respiratory test used extensively in ALS for the assessment of inspiratory muscle strength. In this study, we aimed to investigate the role of SNIP at baseline to predict PEG placement in ALS. Data from a clinical incident cohort of 179 ALS cases attending the multidisciplinary ALS unit of the University of Bari between April 2006 and December 2012 were retrospectively analysed. At baseline, patients underwent detailed neurological, nutritional and respiratory assessments, including measurements of SNIP and forced vital capacity (FVC). Patients were therefore followed up approximately every three to six months until they were able to attend the centre. The censoring date for the survival analysis was 15 April 2014, with PEG placement as the main outcome. Cox proportional hazard regression models were used to examine the association between SNIP and PEG placement, adjusted for possible confounders. During the follow-up period, 75 participants (42%) received PEG implant. PEG placement was more frequent (57% vs. 31%; *p* = 0.001) and earlier (after 11.6 ± 14.0 months from the first visit, vs. 23.3 ± 15.5 months; *p* < 0.0001) in the group of patients with baseline SNIP ≤ 40 cm H_2_O. Baseline SNIP was a predictor of PEG placement even after correction for multiple potential confounders (HR 0.98; 95% CI: 0.96–0.99; *p* = 0.02). To conclude, the present study showed that SNIP at baseline is an early indicator of disease progression and therefore of the need for enteral nutrition in ALS.

## 1. Introduction

Amyotrophic lateral sclerosis (ALS) is a rapidly progressive neurodegenerative disease mainly characterized by motor-neuron degeneration in the spinal cord, brainstem and motor cortex, which leads to progressive muscular atrophy, paralysis, speech and swallowing disturbances, as well as respiratory dysfunction [1]. Death is usually due to respiratory failure and occurs typically 2–3 years after diagnosis, although a much higher (up to two decades) and a much lower (less than 1 year) disease duration has been reported in some cases [2,3].

Risk of malnutrition is elevated in ALS, and presence of malnutrition and weight loss has been related to survival, even in an early stage of the disease [4,5]. Dysphagia is one of the main determinants of malnutrition in ALS [6], although hypermetabolism, present in more than 50% of ALS patients, is also responsible of progressive weight loss [7].

Percutaneous endoscopic gastrostomy (PEG) is a safe and effective procedure for enteral feeding in ALS [8], with a clear overall significant effect on increasing ALS survival, regardless of the site of onset of the disease, and especially if forced vital capacity (FVC) is ≥50% of the expected value, at PEG insertion [9]. PEG is generally better tolerated than nasogastric tube for long-term enteral feeding [10]. Current guidelines for nutritional management of patients with ALS recommend PEG implant when nutritional status deteriorates with a weight loss of more than 10% of subject’s usual weight, and before FVC falls below 50% of the predicted weight [11,12,13]. However, several studies proposed an earlier use of enteral nutrition during the disease course, since higher BMI and higher albumin levels at the start of enteral nutrition have been related to longer survival [8,14,15], as well as better respiratory performance (FVC ≥ 60%) [9].

Nasal Inspiratory Pressure during a Sniff (SNIP) is a valid respiratory test, extensively used in ALS for the assessment of the inspiratory muscle strength [16]. It can be a good indicator of respiratory muscle weakness in the early phase of the disease, even when FVC is normal [17,18], or when patients with bulbar involvement are not able to correctly perform spirometry [19]. Several studies have shown that SNIP can provide prognostic information and is a better predictor of tracheostomy or death than FVC [20,21]. However, there are no studies to date investigating the role of SNIP as a predictor of starting enteral nutrition in ALS.

In the present study, we aimed to investigate the role of baseline values of SNIP as a predictor of PEG placement in ALS.

## 2. Materials and Methods

### 2.1. Study Design and Study Population

#### Retrospective Observational Study

The study is based on a retrospective analysis of a clinical cohort of patients with ALS attending the Centre for Neuromuscular Diseases at the University of Bari “Aldo Moro”. All the patients receiving a new (incident) diagnosis of ALS according to El Escorial criteria [22], between April 2006 and December 2012, were included in this study.

### 2.2. Procedures of Assessment

As part of routine clinical assessment and standard of care at the Centre, all patients underwent a baseline neurological examination by ALS-expert neurologists. The neurological examination was focused, in particular, on the identification of upper motor neuron (UMN) and lower motor neuron (LMN) signs, and their distribution over several body regions. Functional status was assessed using the revised ALS Functional Rating Scale (ALSFRSr) [23]. Nutritional assessment included measurement of weight and height, and calculation of the body mass index (BMI) according to the formula BMI = weight, kg/(height, m)^2^. Body weight was measured with an electronic floor scale SECA^®^ in those patients who could stand independently on the scale, or with a chair scale for those patients who could not stand. Height was measured using a stadiometer incorporated in the floor scale, or collected from the patient’s identity card, if the patient was not able to stand. Malnutrition was defined based on a value of BMI ≤ 18.5 kg/m^2^ [24]. The Charlson Comorbidity Index (CCI) [25] was also calculated for each participant.

Respiratory tests were performed by experienced technicians under the supervision of a pneumologist. Spirometry was performed with the patient in the sitting position, using a spirometer PK Morgan Ltd, Gillingham, UK. The equipment was calibrated using a 3-L syringe and the analysis was performed according to the American Thoracic Society (ATS)/European Respiratory Society (ERS) Guidelines [26]. For FVC, the best of three reproducible values, expressed as a percentage of the predicted value, was taken into account. To overcome air leakage from the mouth, a full-face mask was adopted for patients with bulbar impairment. The sniff test was performed with a MicroRPM-Respiratory Pressure Meter. The patient was seated on a chair and the plug-catheter was inserted in one nostril, while the other extremity of the catheter was connected to a pressure transducer. SNIP was measured in one nostril during a maximal sniff, while the other nostril was closed with a sealing plug. Patients were asked to breathe normally from their nose, with the mouth closed, and to perform at least five maximal sniffs, every 30 s. The highest of five results was recorded. The following criteria were used to select a suitable sniff: (a) a pressure curve showing a regular upstroke and a sharp peak and (b) a total sniff duration of less than 0.5 s [27]. The pressure was expressed in cmH_2_O. Patients were stratified according to a SNIP cut-off value of 40 cmH_2_O, the same was proposed by the EFNS Task Force as one of the criteria for starting NIV [11,20]. Patients also underwent an arterial blood drawn for measuring partial pressure of oxygen (PaO_2_) and carbon dioxide (PaCO_2_).

Patients were routinely evaluated approximately every three to six months until they were able/available to attend an in-person visit at the centre. All the baseline assessments were also performed during each follow-up visit. 15 April 2014 was used as the censoring date for this study.

### 2.3. Nutritional Management

Nutritional counselling (chin-tuck manoeuvre, modification of food and fluid consistency, use of nutritional supplements) was used as the first approach to manage mild dysphagia. When the suggested measures were no longer effective, enteral feeding with PEG was suggested as a sole source of nutrition. The main clinical indicators of PEG placement were (a) the presence of nutritional status deterioration, with dysphagia and a weight loss of more than 10% of the patient’s usual weight and; (b) FVC above 50% of the predicted value [12,13]. PEG was placed using the Pull technique [28].

### 2.4. Ethics

Our study protocol was approved by the Institutional Review Board of ‘Azienda Ospedaliera Policlinico Consorziale, Bari’, and written informed consent was obtained from all patients.

The present study was conducted according to the World Medical Association’s 2008 Declaration of Helsinki, the guidelines for Good Clinical Practice and the Strengthening the Reporting of Observational Studies in Epidemiology (STROBE) statement [21].

### 2.5. Statistical Analysis

Patients’ baseline characteristics were reported as frequencies (percentages) and mean ± standard deviation (SD). Comparisons between groups were performed using Pearson Chi-square and two sample t tests (or Mann–Whitney U test as appropriated) for categorical and continuous variables, respectively.

For the survival analysis, the overall survival time was defined as the time between baseline visit (date of measurement of the predictor—SNIP), and PEG placement (main endpoint/outcome). For subjects who did not experience the endpoint, overall survival time was defined as the time between the baseline and the date of the last available clinical follow-up. Kaplan–Meier survival curves were estimated for graphical purposes, and survival curves of the two groups were compared using the log-rank test. Univariable and multivariable time-to-event analyses were performed using Cox proportional hazard models to investigate the association between SNIP and time to PEG placement. Another time to event analysis was conducted to investigate the effect of baseline FVC on time to PEG placement. Risks were reported as hazard ratios (HR) along with their 95% confidence interval (CI).

Significance was established at a *p*-value < 0.05. All analyses were conducted with SPSS 22 software.

## 3. Results

One hundred and seventy-nine consecutive patients with ALS have been enrolled in this study. Baseline demographic and clinical characteristics of the study cohort are reported in Table 1.

The mean age was 66.87 (SD: 13.33) years. Fifty-seven percent of subjects were males.

The mean follow-up time was 38.1 (SD: 24.3) months. In the whole sample, mean baseline SNIP value was 49.4 (SD: 27.2) cmH_2_O and mean FVC value at baseline was 80.5% (SD: 27.0). During the follow-up period, 75 of 179 (42%) patients received PEG placement.

Group comparisons between patients with baseline SNIP ≤ 40 (n = 76) and patients with baseline SNIP > 40 (n = 103) are reported in Table 2.

**Figure 1:**
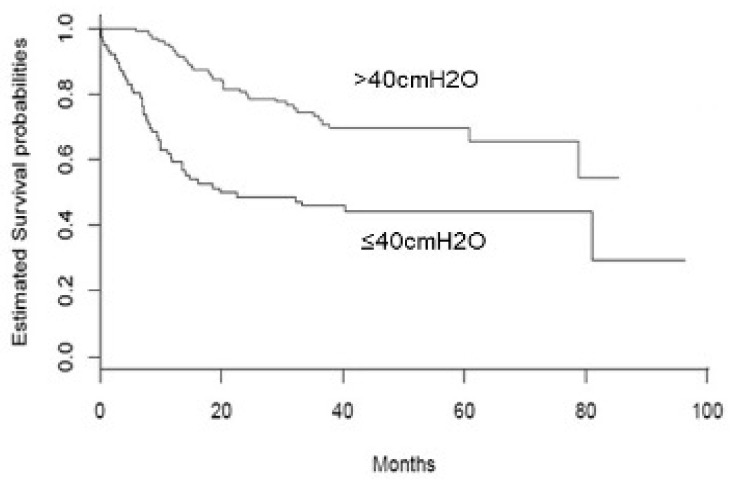
Kaplan–Meyer survival curves of ALS cases stratified according to SNIP value (≤40cmH_2_O vs. >40cmH_2_O).

At baseline, patients with SNIP ≤ 40 cmH_2_O were on average 5 years older (69.6 ± 14 vs. 64.9 ± 12.6 years; *p* = 0.02) and presented lower values of PaO_2_ (85.8 ± 14.9 vs. 92.6 ± 11.8 mmHg; *p* < 0.0001), FVC (63.4 ± 24.9 vs. 92.8 ± 21.2%; *p* < 0.0001), ALSFSRr total score (31.9 ± 8.6 vs. 39.4 ± 5.8; *p* < 0.0001), and of BMI (24.2 ± 5.1 vs. 26 ± 3.7 kg/m^2^; *p* = 0.008). Consistently, PEG placement was more frequent (57% vs. 31%; *p* = 0.001) and earlier (after a mean time of 11.6 ± 14.0 months from the first visit, vs. 23.3 ± 15.5 months; *p* = 0.001) in this group of patients (Table 2). Figure 1 shows Kaplan–Meier survival curves for the main endpoint (PEG placement).

In a time-to-event analysis, higher SNIP at baseline resulted in a lower risk to reach the outcome (HR = 0.98, 95% CI: 0.97–0.99; *p* < 0.0001). An increment of one point of SNIP resulted in a risk reduction of 2%. After adjusting for age, sex, site of onset of the disease, disease duration from onset, baseline respiratory parameters (PaO_2_, PaCO_2_, SatO_2_), baseline BMI, CCI, and ALSFRSr, baseline SNIP remained an independent predictor of the outcome (HR = 0.98, 95% CI: 0.96–0.99; *p* = 0.02) (Table 3).

Other independent predictors of the outcome were sex (HR males/females = 0.48, 95% CI: 0.25–0.95; *p* = 0.03) and baseline ALSFRSr (HR = 0.95, 95% CI:0.92–0.99; *p* = 0.03).

Conversely, FVC was not an independent predictor of PEG placement (HR = 0.99, 95% CI: 0.98–1.01; *p* = 0.5) when investigated in a multivariable Cox proportional hazard model including the same covariates as the previous one (data not shown).

## 4. Discussion

In the present observational study, we found that SNIP, an indicator of respiratory muscle strength, performed at baseline, is an independent clinical predictor of PEG placement. Death in ALS is mainly caused by respiratory failure, resulting from progressive weakness of respiratory muscles [29]. It is well known that patients with ALS, due to dysphagia and hypermetabolism, have negative energy balance, which leads to muscle weakness, fatigue, malnutrition, and death [4,30]. Although in ALS, total daily energy expenditure (TDEE) decreases as disease progresses, it has been demonstrated that energy intake is always lower than TDEE in all the stages of the disease [31], underlying the need of enteral nutritional supplements, even in the early stages. Chronic energy deficiency is in fact likely to have negative consequences for ALS patients by enhancing weakness and fatigue of the remaining innervated muscles and by imposing increased metabolic demands on them to maintain mobility and ventilation [32].

Current guidelines for the management of patients with ALS suggest that enteral nutritional support should be started when weight loss is greater than 10% of the patient’s usual weight, respiratory function is still good (FVC > 50%), and dysphagia is present [12,13]. However, although there are currently no well-established guidelines regarding the right time to start enteral nutritional support [14,32,33], several studies indicate that early PEG placement stabilizes BMI and prolongs survival [8,15,24]. PEG has been successfully used to treat ALS patients with dysphagia since the early ‘90s [33,34], and it has been proven to be effective in the management of malnourished patients, with a minimal morbidity and virtually no acute mortality [12]. Although the AAN ALS practice parameters established that FVC lower than 50% of the predicted value confers a moderate-to-severe risk for a PEG placement [14], there is evidence that the surgical procedure is relatively safe and can also be of benefit in patients with a substantial reduction of FVC [35]. Furthermore, alternative techniques, such as radiologically inserted gastrostomy and per-oral image-guided gastrostomy, have also been demonstrated to be safe and effective in ALS [36], and can therefore be taken into account as appropriate.

Several studies reported that SNIP is a sensitive and specific measure of respiratory dysfunction in ALS [20,21]. SNIP is a non-invasive measure of nasal pressure, and is both a sensitive respiratory test to early detect respiratory muscle weakness and a good parameter of disease progression in ALS [20]. It has been showed that the Sniff test has greater sensitivity and specificity than FVC to predict survival [21,37,38], and to early detect sleep respiratory disorders in ALS patients [39,40]. FVC may indeed not drop until there is severe muscle weakness [37]. Furthermore, SNIP correlates well with invasive and non-volitional tests of diaphragm strength in ALS [41], and it is also used to decide the optimal time to start NIV [39,40,42]. A value of SNIP < 40 cmH_2_0 has been included by the EFNS Task Force, among the proposed criteria for the starting of NIV [11]. This cut-off is highly sensitive (97%) and specific (79%) in predicting mortality within 6 months [11,20]. Furthermore, in a previous study by our group, a shorter survival in ALS cases with SNIP < 43 cmH_2_O was showed [21], and SNIP resulted in being a stronger predictor of tracheostomy/death compared to FVC [21]. In line with these results, in the present study we showed that SNIP at baseline was an independent predictor of the subsequent PEG placement, even after correction for possible confounders, such as age, site of symptoms onset, blood gases, CCI, and BMI value. On the other hand, FCV did not show similar predictive value in the same statistical model. This may be related to the fact that muscle weakness and malnutrition are strictly linked, and SNIP is the first parameter influenced by respiratory muscle weakness, while FVC may not fall until the development of a severe muscle weakness [17,18]. We can therefore argue that a SNIP cut-off value of 40 cmH_2_O can be considered a useful cut-off for mortality prediction, NIV initiation and PEG indication in ALS.

The process of decision making regarding therapeutic interventions in ALS is complex and should take into account factors other than practice guidelines and clinical indication. For example, a recent study showed that sociocultural factors, namely religiousness, personal values, quality of life, and depressiveness, strongly influence decision making regarding NIV, invasive ventilation (IV), or PEG [43]. The more oriented towards traditional and conservative values, the less likely patients are to decide on invasive therapeutic devices (IV, PEG) [43]. However, a better understanding of negative prognostic factors in an early stage of the disease may help clinicians in finding the most appropriate time to start discussion regarding the need of PEG implant. On top of the level of dysphagia, caloric intake, and weight loss, respiratory function is one of the main features of the disease to consider before starting to discuss PEG implant with the patient. Moreover, the use of respiratory support at the time of implant is an important characteristic. The use of NIV at the time of PEG placement has in fact been reported as a negative prognostic factor for survival after PEG [44].

Several limitations must be considered for this study. Firstly, the study cohort is clinical based and not population based, and thus may be influenced by referral bias [45]. Secondly, the analysis has been conducted retrospectively. Furthermore, it was not possible to collect data on daily food intake and energy expenditure in order to have a comprehensive nutritional assessment of the included subjects and adjust for other possible confounders [24].

## 5. Conclusions

In summary, our study conducted in a large cohort of ALS cases with neurological, nutritional and respiratory assessments, underlines the role of SNIP at baseline as a strong predictor of PEG placement in amyotrophic lateral sclerosis. SNIP, as an early indicator of respiratory muscle weakness, can have an important role in early identification of patients for which enteral nutrition is indicated. This may help clinicians to recognize the right time to discuss the need of enteral feeding and gastrostomy indication with patients and caregivers. Further longitudinal studies are needed to confirm these preliminary observations.

## Figures and Tables

**Table 1:** Demographic and clinical characteristics of the whole sample (n = 179).

Variable (n)	M ± SD, or n (%)
Age (179) (years)	66.87 ± 13.33
Sex M (179)	102 (57.0)
Type of Onset (179)	
– Spinal	128 (71.5)
– Bulbar	51 (28.5)
CCI (179)	
0	41 (22.9)
1	96 (53.6)
2	29 (16.2)
3	10 (5.6)
4	3 (1.7)
Time onset—1st visit (179) (months)	24.3 ± 12.9
ODI (179) (months)	15.7 ± 11.6
Time diagnosis—1st visit (179) (months)	8.7 ± 8.2
Follow-up time (PEG or last clinical follow-up) (179) (months)	38.1 ± 24.3
Time 1st visit—PEG (n = 75) (months)	16.6 ± 15.7
PEG(179)	
Yes	75 (41.9)
No	105 (58.1)
PaO_2_ (179) (mmHg)	89.7 ± 13.6
PaCO_2_ (179) (mmHg)	39 ± 5.4
pH (179)	7.3 ± 0.8
SatO_2_ (179) (%)	97.1 ± 1.7
ALSFSRr (127)	36.3 ± 8
SNIP (179) (cmH_2_O)	49.4 ± 27.2
SNIP categories (179)	
>40 cmH_2_O	103 (57.5)
≤40 cmH_2_O	76 (42.5)
FVC (174) (%)	80. 46 ±27
BMI (179) (kg/m^2^)	25.3 ± 4.4

CCI: Charlson comorbidity index. ODI: onset-diagnosis interval. PEG: percutaneous endoscopic gastrostomy. ALSFRSr: amyotrophic lateral sclerosis functional rating scale revised. SNIP: sniff nasal inspiratory pressure. FVC: forced vital capacity. BMI: body mass index.

**Table 2:** Comparison between ALS groups stratified according to SNIP cut-off value (≤40 cmH_2_O).

Variables (n)	SNIP ≤ 40 cmH_2_O (n = 76) M ± SD, or n (%)	SNIP > 40 cmH_2_O (n = 103) M ± SD, or n (%)	*p*-Value
Age (179) (years)	69.6 ± 14.0	64.9 ± 12.6	**0.02**
Sex (179)			0.25
M	39 (51)	63 (61)	
F	37 (49)	40 (39)	
Type of onset (179)			0.2
Spinal	26 (34)	25 (24)	
Bulbar	50 (66)	78 (76)	
CCI (179)			0.7
0	18 (24)	23 (22)	
1	37 (49)	57 (59)	
2	14 (18)	15 (15)	
3	6 (8)	4 (4)	
4	1 (1)	2 (2)	
Time onset—1st visit (179) (months)	23.2 ± 12.7	25.2 ± 13.0	0.3
ODI (179) (months)	15.0 ± 11.0	16.1 ± 12	0.5
Time diagnosis—1st visit (179) (months)	8.1 ± 7.3	9.09 ± 8.91	0.4
Follow-up time (PEG or last clinical follow-up) (179) (months)	31.8 ± 28.2	42.7 ± 19.9	**0.004**
Time 1st visit—PEG (75) (months)	11.6 ± 14.0	23.3 ± 15.5	**0.001**
PEG (179)			**0.001**
Yes	33 (43)	71 (69)	
No	43 (57)	32 (31)	
PaO_2_ (179) (mmHg)	85.8 ± 14.9	92.59 ± 11.8	**<0.0001**
PaCO_2_ (179) (mmHg)	39.8 ± 6.2	38.4 ± 4.6	0.1
pH (179)	7.4 ± 0.82	7.3 ± 0.8	0.9
SatO_2_ (179) (%)	96.8 ± 2.1	97.3 ± 1.4	0.1
ALSFSRr (127)	31.9 ± 8.6	39.4 ± 5.8	**<0.0001**
FVC (174) (%)	63.4 ± 24.9	92.8 ± 21.2	**<0.0001**
BMI (179) (kg/m^2^)	24.2 ± 5.1	26.0 ± 3.7	**0.008**

CCI: Charlson comorbidity index. ODI: onset-diagnosis interval. PEG: percutaneous endoscopic gastrostomy. ALSFRSr: amyotrophic lateral sclerosis functional rating scale revised. SNIP: sniff nasal inspiratory pressure. FVC: forced vital capacity. BMI: body mass index. All significant p-values (p < 0.05) are in bold.

**Table 3:** Multivariate COX proportional hazard model of predictors of PEG placement.

	HR	95% CI	*p*
Sex (males vs. females)	0.48	0.25–0.95	**0.03**
ALSFRSr	0.95	0.92–0.99	**0.03**
SNIP	0.98	0.96–0.99	**0.02**

Other non-significant predictors were age, site of symptoms onset, disease duration from onset, respiratory parameters (PaO_2_, PaCO_2_, SatO_2_), BMI, Charlson Comorbidity Index. **ALSFRSr:** amyotrophic lateral sclerosis functional rating scale revised. **SNIP:** sniff nasal inspiratory pressure. All significant *p*-values (*p* < 0.05) are in bold.

## Data Availability

All data are available upon request to the corresponding author.
